# The social relations of health care and household resource allocation in neoliberal Nicaragua

**DOI:** 10.1186/1472-698X-10-9

**Published:** 2010-05-22

**Authors:** Laura E Tesler

**Affiliations:** 1 Department of Social and Behavioral Sciences, University of California, San Francisco, San Francisco, CA 94143, USA

## Abstract

**Background:**

With the transition to neoliberalism, Nicaragua's once-critically acclaimed health care services have substantially diminished. Local level social formations have been under pressure to try to bridge gaps as the state's role in the provision of health care and other vital social services has decreased. This paper presents a case study of how global and national health policies reverberated in the social relations of an extended network of female kin in a rural community during late 2002 - 2003.

**Methods:**

The qualitative methods used in this ethnographic study included semi-structured interviews completed during bi-weekly visits to 51 households, background interviews with 20 lay and professional health practitioners working in the public and private sectors, and participant-observation conducted in the region's government health centers. Interviews and observational field notes were manually coded and iteratively reviewed to identify and conceptually organize emergent themes. Three households of extended kin were selected from the larger sample to examine as a case study.

**Results:**

The ongoing erosion of vital services formerly provided by the public sector generated considerable frustration and tension among households, networks of extended kin, and neighbors. As resource allocations for health care seeking and other needs were negotiated within and across households, longstanding ideals of reciprocal exchange persisted, but in conditions of poverty, expectations were often unfulfilled, exposing the tension between the need for social support, versus the increasingly oppositional positioning of social network members as sources of competition for limited resources.

**Conclusions:**

In compliance with neoliberal structural adjustment policies mandated by multilateral and bilateral agencies, government-provided health care services have been severely restricted in Nicaragua. As the national safety net for health care has been eroded, the viability of local level social formations and their ability to respond to struggles collectively has been put at risk as well. Bi-lateral and multilateral agencies need to take into account local needs and demands, and implement policies in a manner that respects national laws, and protects both the physical and social well-being of individuals.

## Background

Peter Uvin defines structural violence as "a condition in which the poor are denied decent and dignified lives because their basic physical and mental capacities are constrained by hunger, poverty, inequality, and exclusion," owing to political, economic and social factors [1]. Concordant with economic policies that routinize and legitimize political, social and health disparities, he argues, structural violence also undermines social stability by contributing to the reduction of people's self-respect, the social marginalization of certain segments of the population, and the disruption of community-level social relations. Drawing from Uvin's analytical framework, this article presents a case study of how the impacts of contemporary global and national health policies reverberated in the social relations of a Nicaraguan community in 2002 - 2003.

The impact of neoliberal structural adjustment policies (SAPs) and international development interventions in the sphere of health care has been well-documented from a macro-level perspective in the development aid literature [2-4]. However, there remains a paucity of ethnographic analysis that incorporates the health care seeking experiences of those whose daily lives have been dramatically affected by these policies. Health care seeking is a complex, non-linear process embedded within other micro and macro level domains of a social system. It thus requires the holistic analytical approach offered by ethnographic methods. Ethnographic research is also vital for gaining insights into delicate issues, such as painful decisions regarding resource allocations among family members, which cannot be obtained through surveys.

Noting the challenges of demonstrating the specific ways that structural violence is connected with personal suffering, Farmer has argued for analyses that "embed individual biography in the larger matrix of culture, history, and political economy" [5]. Using illness as a lens with which to view the social relations of the poor, this paper explores how structural violence weakened social relations within and across three households of extended kin, as the government safety net for health care steadily eroded, and people's ability to maintain social support through reciprocal exchange relations diminished. As they struggled to negotiate resource allocations for health care seeking and other needs, conflicts ensued between an ethos of solidarity and cooperation, and demands for individual competition and self-preservation. I argue that the anguish expressed by study participants over their inability to comply with social norms, or to rely on others to meet their obligations, indexes an additional form of violence and indignity suffered by the populace due to macro-level policies that have prioritized the state's economic obligations to bilateral and multilateral agencies over the basic human needs of its citizens.

Beyond the physical hardships of illness, material deprivation confers considerable moral anguish upon the poor. Part of suffering stems from knowing that more could be done for oneself and for others were the resources available. What happens to households when they are in a situation of acute poverty, and one or more individuals become ill, but other members are also in need of resources? What are the tensions that emerge between cultural ideals and economic contingencies, and how are they played out? This ethnographic case study provides an initial foray into these issues.

### Historical context

Currently ranked as the second poorest country in the western hemisphere, after Haiti, Nicaragua suffers from elevated rates of unemployment, child morbidity and mortality, and maternal mortality. Nationally, 48% of the population has been estimated to live in poverty; in the health care arena, 60% have access to health services of poor quality, and 40% have no access to health care services at all [6,7]. Nicaragua makes for a particularly interesting case study of how government health and development policies and programs shifted as a result of changing ideologies and social relations at national and global levels during three distinct historical periods: the authoritarian dictatorship of the Somoza dynasty (1936 - 1979), the socialist revolution of the Sandinistas (1979 - 1990), and neoliberal civilian democratic governance (1990 - Present).

The history of political economy of Nicaragua has been shaped by interacting international and domestic social relations, resulting in the production and reproduction of political, economic and social inequality. From 1936 - 1979, the country was ruled by the Somoza family dynasty. While increases in agricultural production exceeded that of the other Central American nations from 1950 to 1977, most of the benefits were reaped by three large financial groups with ties to foreign banks. Dominating one of these groups, the Somoza family embezzled much of the development aid and eventually amassed an estimated fortune of $500 million, including up to 15% of the country's land. Rather than using development aid to diversify crops, state-led production intensified coffee and cotton cultivation, rendering the economy vulnerable to fluctuating world market prices, displacing greater numbers of peasants from their lands, and increasing unemployment rates by replacing human labor with machinery. Cotton cultivation, moreover, encroached on grain production, transforming the country from a net exporter to a net importer, thus undermining its food security [8,9]. The Somozas contained popular uprisings and elite dissent through extreme measures of repression executed by the National Guard. By the end of the 1970s, the regime had taken the lives of an estimated 50,000 people - in a country whose population totaled just under 3 million at that time - culminating in the 1979 revolution led by the Sandinista National Liberation Front (FSLN), a military movement of students, workers and peasants [9-11].

Shortly after the FSLN assumed power, class tensions and other competing interests reemerged. Efforts to redistribute resources through agrarian reform, food subsidies, housing, mass literacy campaigns, and universal access to health care generated much popular support, but threatened the privileged status of members of the middle and upper classes. More broadly, the revolution "had raised expectations that it could not meet" [12], as competing interests within and across classes, as well as gender and ethnic issues, were not easily reconcilable through legislative changes and a socialist mixed economy that preserved private property and production [10,12,13]. These internal conflicts articulated with the breakdown of external relations with the United States. Viewing the Sandinista government "as an extension of Soviet communism", the Reagan administration terminated its development aid and trade relations, led an international trade embargo, and provided training and support for a counterrevolutionary army aimed at overthrowing the FSLN [13]. By the end of the 1980s, a beleaguered populace voted the pro-U.S. National Opposition Union (UNO) coalition to power, and democratic elections have been held for the past 16 years [13,14].

Loker defines neoliberalism as "a theory of political economy that claims the market is the most efficient mechanism for the distribution of goods, and that state interference with the workings of the market and capitalist production should be minimized" [15]. While the utilization of coercive measures to repress dissent has substantially diminished, structural violence has increased, and in the process, social stability has been undermined. In practice, neoliberal structural adjustment policies, in Nicaragua and elsewhere, have reduced the size and power of the ministries of education, health, agriculture, and social services, promoting privatization of these sectors. While the SAPs required by bilateral and multilateral donor agencies may have been implemented with the intention of helping to stabilize the economy, unemployment and under-employment rates in Nicaragua actually rose during the 1990s, as did the disparity of wealth and the nation's internal and external debt [13,16,17]. In 2004, workers' monthly wages averaged between a low of $41.50 in the agricultural sector and a high of $98.00 in the banking sector, whereas the average cost of a "basic basket" of goods was $157.40 [14]. From 1990 to 2007, the nation's ranking in the Human Development Index fell from 60 to 110 [18]. After nearly two decades of structural adjustment programs, the benefits have yet to accrue to the majority of the Nicaraguan populace.

### Historical shifts in health care services

During the Somoza dictatorship, medical care was inaccessible to a majority of the population, especially the poor living in rural areas. In the 1970s, just 28% of Nicaraguans "had regular access to health care" and "90% of health resources went to 10% of the population" [19]. In 1979, having identified development of the health sector as a key priority, the new Sandinista regime created the National Unified Health System. Over the next few years, the number of clinics more than tripled, with the establishment of new facilities in rural areas taking precedence, and accessibility to health care services extending to cover 70% of the population. Declaring health care to be a fundamental right of the population, the government promised free care in government facilities, and the number of visits to physicians per capita more than tripled by 1983, as did surgeries and prescriptions filled [20].

Following the regime change in 1990, under the mandates of the United States Agency for International Development (USAID), the World Bank, and other donor agencies, the Nicaraguan government implemented a series of structural adjustment programs (SAPs) intended to stabilize the economy, in part through the downsizing of health care and other vital social services formerly provided through the public sector. SAPs quickly resulted in significant decentralization, privatization, a reduction of government personnel, and a reduction in spending in the health sector. From 1992 to 1996, real health spending was cut by more than 12% [21].

Starting in 1991, primary and secondary health facilities were organized into 17 regional jurisdictions to be administered through a Local Integrated Health Systems model (SILAIS) rooted in a Pan American Health Organization (PAHO) design. While each regional SILAIS was charged with responsibility for the provision of clinic and hospital services, the majority of the health budget continued to be under the control of the central Ministry of Health, as were many aspects of policymaking, hence actual local-level decision-making capacity was limited [21,22]. In 1993, the Social Security Institute was once again made directly responsible for the health care of workers in the formal sector, and its services in turn were privatized as it contracted outpatient services to private insurance companies, while private hospital wards were established within MINSA for the in-patient services. Each local SILAIS was granted authority to sell services to INSS and private purchasers, ostensibly to use the funds generated to subsidize its other activities; instead, they were reinvested in the private wards, thus improving the quality of care only for patients with Social Security or who could afford to pay out of pocket [21]. While only approximately 5% of the population was covered through INSS at the time, 36% of the entire national public health budget went to subsidizing their private insurance providers in 1995 [23].

Significantly, much of the privatization, decentralization and increases in costs incurred to patients that occurred was *not *planned, but arose instead as indirect consequences of health policies and economic activities. Many health facilities began to collect user fees as their budgets shrank and supplies dwindled [20,21]. Although user fees were banned in 1998, the law has not been enforced [24]. Due to poor coverage by the public sector, private providers of ambulatory services increased from 17% in 1993 to 33% in 1998 [24]. In addition, public sector physicians whose wages were decreased through inflation in accordance with IMF guidelines, resorted to augmenting their incomes by practicing in the private sector during the evenings and weekends [20,21].

By 1995, 36.5% of health costs were being covered directly by households, a greater percentage than that paid by the government [23]. In 2001, the amount of funds designated to reduce chronic malnutrition in children under age 5 was equivalent to just US $1.37 per child; by 2003, the government per capita health expenditure was the least of any Central American nation [25]. Medications have constituted greatest out of pocket costs for households, consuming 47.2% of household health expenditures in 2001 [24]. Although free or subsidized drugs are available in the public sector, inventories in government health centers and hospitals have been inadequate due to the distribution systems being overly centralized and poorly managed. As a result, 55% of the population lacked access to medications through public sector services, and the medications available through the private sector were prohibitively expensive [24,26].

Public expenditures on health care have been further exacerbated by the government's poor budget management and corruption, contributing to medical supply shortages throughout the public health system [27]. In 2005, due to the low quality of health services, lack of medications available in public sector health facilities, the high cost of out of pocket expenses, and distance from the nearest health facility, two-thirds of the population were reported to forego seeking care when ill [28]. In the arena of health care, national poverty has thus been manifest both in the lack of resources available in the Nicaraguan government health facilities, and in the lack of resources available in individual households.

### Previous research on structural violence to household social relations in Nicaragua

Political economic forces, including structural adjustment policies, negatively impact local systems of reciprocal exchange, as certain patterns of resort may be less available due to exacerbations in poverty conditions [29]. When one form of state or social support diminishes or disappears, people may actively seek out others. However, as research in Guatemala and Rwanda has shown, even as new forms of support emerge, or already existing forms increase in importance, extended periods of political and structural violence ultimately undermine the social relations of communities [1,30].

To be certain, Nicaragua's economic instability and wealth disparities began well before the advent of neoliberal policies [8,9,20]. Qualitatively, however, the impact of these policies, particularly the downsizing of public sector services, has been dramatic for local systems of reciprocal exchange. Prior scholarship on Nicaragua has described the rise of kin networks and community-based forms of collective procurement and sharing of resources in response to the war and prolonged economic crisis during the 1980s, and extending into the early 1990s [10,13,31-33]. Female-headed households in Managua, whose number increased during the periods of war and economic crisis of the 1970s - 1980s, became especially active in resource circulation through fictive kin networks [10]. A study of household economics in a Managuan neighborhood during the mid-1990s, however, described the replacement of group-based survival strategies with the emergence of "non-cooperative" households, accompanied by an ethos of mistrust among neighbors, and a refusal to share resources both within and across households [34]. Other scholars noted the undermining of local level social relations as cooperatives have been replaced by microenterprises which put neighbors in competition with one another, increases in domestic violence, and increases in youth delinquency, gangs and crimes [13,35].

### Case study: San Rafael

The data presented in this case study are drawn from ethnographic fieldwork conducted from November 2002 through October 2003 in San Rafael (a pseudonym), a predominantly rural municipality located about 20 miles outside of Managua, with a population of about 40,000 [36]. The majority of people in San Rafael, like those in the rest of the nation, live in poverty. According to data provided by the Mayor's office, an estimated 28% of the economically-active population of this community was unemployed, while another 22% could only obtain part-time work, and most households earned far less than $150 a month, the estimated cost of a "basic basket" of consumer subsistence goods [37,38]. Among those households participating in the study, 53% reported delaying or not seeking treatment for one or more illness events because they could not afford the costs [36]. Previous residence in this region as a Community Health Promoter with Peace Corps during the mid-1990s provided the basis of my initial concern with the subject matter addressed here.

During the fieldwork period, reciprocal exchanges among residents of San Rafael in the form of food, medicines, clothing, temporary shelter, and so forth, occurred within the context of mundane everyday life, and as part of more elaborate rituals in conjunction with holidays and times of crisis. As Lancaster observed in Managua during the 1980s, such exchanges occurred within the context of a "gift economy", framed as acts of friendship or affection as opposed to barter [10]. Acts of giving both large and small, moreover, were explicitly linked to people's moral identities as members of their local community and as citizens of the Nicaraguan nation-state. To this end, people spoke with pride as they described in the present study how Nicaraguans were known for their exceptional generosity. Increasingly, however, reciprocal relations had become difficult to sustain. In addition to accusing others of stinginess or of taking advantage, people frequently protested in self-defense that they could not help others when they did not even have enough for themselves.

Notably, several study participants recalled mutual assistance among relatives and neighbors as having been stronger during both the dictatorship and revolutionary eras, as was the overall stability of the social order. Not unlike the collective efforts noted in the case studies conducted by scholars during the 1980s and early 1990s, for all the hardships residents of San Rafael vividly remembered experiencing during these periods, many also emphasized how they had helped each other. Even neighbors experiencing strained relations during the study period recounted tales of past cooperation with one another through both open and clandestine exchanges of scarce commodities.

Nevertheless, political violence experienced during the dictatorship and Contra war, the prolonged economic crisis, and the ongoing erosion of vital public sector services from 1990 onwards generated strife among households, networks of extended kin, and neighbors. Following several years of structural adjustment programs that substantially diminished the material resources that could be shared, the emotional dimension of exchange relationships had been weakened as well. Conflicts emerged as cultural ideals pertaining to reciprocal exchange relations and other obligations could not be met. Individuals found it increasingly difficult to fulfill their obligations to share, or to rely on others to do so. One of the most politically and socially charged tensions to arise from conflicts between cultural ideals and material realities within and across households in San Rafael pertained to the allocation of responsibility for health care, the focus of this case study.

## Methods

From November 2002 - January 2003, I conducted background interviews with 20 lay and professional health practitioners working in the public and private sectors, and conducted participant-observation in the region's government health centers, a private pharmacy, and the study site neighborhoods as I accompanied health center staff during their vaccination campaigns, mosquito abatement projects, and pregnancy monitoring activities. I then administered a baseline survey to 214 households, to derive a purposive, stratified sample of 53 households to yield "information-rich" cases for in-depth analysis [39]. One-third of the households selected for the sample were very low income, one-third were low income, and the remainder were lower middle income. Each household had at least one child under the age of six, and 28 households had kin ties to at least one other household participating in the study, facilitating an examination of inter-household social relations.

From March - September, 2003, I made bi-weekly visits to each household, and conducted open-ended, semi-structured interviews with primary caregivers and other household members on household health statuses, illness symptoms, health care seeking activities, reasons for adherence and delays, inter- and intra-household social relations, and changes in the availability of economic resources that occurred during the fieldwork period, such as variations in income-generating opportunities, additions or losses of income-generating household members, or the receipt of remittances from household members seeking work in other parts of the country or in Costa Rica. Following a detailed review and completion of an itemized summary of the field notes and bi-weekly interviews, I concluded that data saturation had been reached based on the lack of newly emerging types of illnesses or health care seeking events, the lack of expansion to the range of social relations or economic circumstances observed in study households, and the cessation of novel responses to the bi-weekly interview questions.

Ethical approval for the study was obtained from the University of Arizona Human Subjects Protection Program. Prior to being interviewed, all participants were informed verbally and in writing that their participation was voluntary, that they could end their participation at any time, and that they could choose not to answer any questions. Participants provided written consent. No economic incentive was offered for participation in the study. Approximately 10% of those invited to participate in the study declined, citing lack of economic compensation or lack of time as their reasons. Of the 53 households that initially agreed to participate in the ongoing bi-weekly visits, two withdrew from the study due to lack of time. To help protect their anonymity, pseudonyms have been assigned to the study site and study participants.

Audio-recording interviews was not possible because the recording device was both a source of distraction and discomfort for study participants. I was, however, able to take extensive written notes, and was accompanied by field assistants who served as a second set of eyes and ears. All interview and field note observations were typed up at the end of each day while events and conversations were still fresh in my mind. I sought clarification of gaps or ambiguities in the notes, and discussed preliminary interpretations with both my field assistants and during subsequent visits with study participants. Interviews and observational field notes were manually coded and iteratively reviewed to identify and conceptually organize emergent themes, which were organized into tables. Three households whose members comprised an extended family were selected from the larger sample to examine as an in-depth, ethnographically rich case study for this paper. I selected these three because 1) the issues addressed at the level of each household and in their inter-household social relations shared much in common with the larger study sample and 2) they were part of the largest kin network included in the sample (totaling 5 households), providing a greater number of vantage points to explore for the analysis on social relations. While the broad spectrum of circumstances and characteristics of the entire study sample cannot be fully encompassed by a single extended family, this family's experiences with inter-related economic hardships, health care seeking issues and tensions in social relationships were resonate with the majority of study households.

This case study focuses on three households of extended female kin. In Nicaragua, women bear the onus of responsibility for the *household production of health *[40], in their roles as mothers, grandmothers, and increasingly, as income-earners. In conjunction with examining how conditions of poverty have undermined people's ability to fulfill cultural ideals pertaining to social support within and across households, I therefore make central the lived experiences and social relations of women. In the following sections, I examine how macro- and micro-level resource scarcities affected the social relations of health care seeking within and across the households of Doña Elisabeth, her daughter Susana, and her daughter-in-law Mariluz.

## Results

### Doña Elisabeth: "We are slaves with these illnesses"

Doña Elisabeth, age 38, lived with her 51-year-old husband, Don Alfonso, their three teenage children, a son-in-law, and two grandchildren in a small house made of rustic wood planks and a dirt floor. Doña Elisabeth possessed a third grade education, and her husband a fifth grade one. None of her children had graduated from secondary school, and her older three daughters had become pregnant during their mid-teens. At any given time throughout the study, the majority of her household, including herself, suffered from multiple ailments.

Doña Elisabeth's teenage son and youngest daughter had little energy, chronic stomach ailments and headaches. Her son had to repeat his second year of high school, and Doña Elisabeth worried that both teens were going to be expelled because of their low grades. Attributing their poor academic performance to their poor health, Doña Elisabeth suspected they suffered from anemia and parasites, but she and her husband were unable to purchase anything other than mild analgesics such as acetaminophen. However, since the health center charged for exams for teenagers, and the visit would require them to miss school, she did not take them there.

Doña Elisabeth's husband, in turn, experienced chronic stomach ailments, muscle aches, and like most household members, had a skin fungus on his feet. At various points over the course of the study, Doña Elisabeth speculated that his stomach troubles were due to parasites, stress over the family's circumstances, a kidney infection, malnourishment, or perhaps his alcohol consumption. He took an over-the-counter analgesic for pain but did not seek treatment for the other ailments.

Doña Elisabeth further worried about her 17-year-old daughter, Arlen. A mother of two, Arlen suffered from nausea, headaches, and pains originating in the "womb," as well as a lump in her breast, but had not sought care for herself. Arlen's husband occasionally took pills for his asthma, but did not treat his other ailments since the little money he earned from selling fruit in Managua went to covering his family's food and the children's health care. As with most study households, the youngest children were prioritized for health care seeking activities and expenditures. Even then, Doña Elisabeth fretted that her grandsons, frequently afflicted with respiratory ailments and parasites, were not receiving adequate treatments, since the health center services were limited; while children under age five were officially identified as a priority population for government health services, the region's health centers possessed limited inventories of medications, and the household did not possess sufficient funds to purchase medicines in the private sector except in cases of emergency.

Doña Elisabeth and her family had a small parcel of land to cultivate limited subsistence crops, as well as fruit that her husband sold in Managua. Income from the fruit sales generated very little cash, however, and most of it went to additional food needs, utilities and the younger teenagers' high school expenses, leaving little left over for health care: "The majority of the house is very sick right now. And there is no money to cure ourselves. We are slaves with these illnesses."

What does it mean to be rendered a "slave" by illness? For Doña Elisabeth and her family, illness could be understood as dominating their lives and livelihoods. As she went on to explain, the national government did not offer the means for escape, nor could her family buy their freedom: "In the health center...we go there and they do not do anything. And the men are without work." Even death offered no relief: "My husband and I worry but we also joke about our health problems and how we are going to cover our funeral costs if we die. My husband recently joked that he will be buried in a rice sack because there will not be any money to buy the wood for a coffin."

Doña Elisabeth further discerned a state of entrapment due to a vicious cycle in the relationship between health and economics: "Health is first but at times we abandon it for our work. The problem is that one can't work well or at all if one is sick, but neither can one take time off from work to treat oneself." To be vulnerable to illness owing to economic constraints was the norm - both because people were unable to avoid getting sick, and because they lacked the money to get better.

During our first interview, when I asked Doña Elisabeth to recall the last time she had visited the local government health center, she replied, "To be honest and frank with you, not since the decade of the 80s. Since then, I no longer know it." Like many residents of San Rafael, Doña Elisabeth believed that currently, the health centers mainly provided services for children, and even for them, the services were limited: "They are only for children," she explained, "but we still have to seek medications for them elsewhere. The health center only provides prescriptions for their infections." Doña Elisabeth recalled that during the Sandinista era, health centers had provided medications for people of all ages, as well as shampoos to treat lice and skin infections (nearly everyone in her household and a majority of the study sample had a skin infection), and how she had received vitamins and extra rations of food and milk from the health center during her pregnancies. Witnessing the lack of comparable prenatal services for her daughters, and the reduction of treatments being offered to her young grandchildren, she felt that the health centers had since become nearly hollow structures:

The health centers exist to support the population, but here they have been liquidated. There are no medications. Before, treatment arrived, but no longer. The children go to the center and return with the same problem. Before, children had low fevers, but now they are high because of the lack of treatments available. Now there is no treatment for anyone.

Doña Elisabeth's declaration of the health center's purpose - to support the population - was in one respect a moral discourse. That even children, widely viewed as the most vulnerable segment of the population, could not receive the treatment they needed, seemed incomprehensible to her. However, her statement was also grounded in law; to date, the Nicaraguan nation is still under constitutional mandate to provide health services to the populace. Doña Elisabeth, like many study participants, believed that the economic situation of her household, the wider community, and the nation was holding them back in achieving good health: "*La salud de aquí es bastante atrasada. No hay dinero, somos pobres*. Health here is very much behind. There is no money, we are poor." The word "*atraso*", which means both "behind" and "delay," was frequently used among study participants to describe the general economic situation of the country, but also extended into a broader discourse on "progress" and "development". When I asked Doña Elisabeth to describe the economic situation of her community, she again related it to health, this time in the context of nutrition: "This is a difficult theme. *No hay*. There is nothing. The homes are badly built because there is nothing. And in nutrition we are low, too low. We cannot advance in order to buy food, not in the quantity we need...there are no economic resources."

Although Doña Elisabeth implicated the state and international donor agencies in her discussion of her family's and fellow citizens' health and economic troubles, she also struggled to comply with the expectations she had for herself and others to continue to seek care no matter what constraints might exist. Although she had initially reported that she had not been to local government health center since the 1980s, she subsequently noted that she did go there a couple of years earlier and was diagnosed with ovarian cysts. An auxiliary nurse there helped her to obtain a low-cost treatment through a non-governmental organization's (NGO's) clinic located in Managua, the nation's capital. Her last two follow up visits showed good results, but she began to experience symptoms again in late 2002. Since the government health center had been lacking the lab materials to perform Pap smears for the past few months, the same nurse offered to write a referral for her to return to the NGO clinic and have a test done for a discounted price of 80 *cordobas *(approximately US $5.00). Doña Elisabeth initially expressed intent to visit the clinic and avoid *el abandono *(abandonment): "Many families give up, they say it's better to die in their homes rather than search in vain for treatments they can't pay for." The clinic exam fee and transportation costs, however, were equivalent to over two days' worth of food for the entire household. Owing to the lack of finances, the trip was postponed indefinitely.

In addition to her reproductive health issues, Doña Elisabeth experienced chronic episodes of emotion-based ailments known locally as *nervios *(nerves) and *susto *(shock). Well-documented in other parts of Latin America [30,41,42], the locally recognized symptoms for these ailments were somewhat vague and overlapping, including trembling and shaking hands, turning pale, and fluctuating blood pressure. The etiology of *nervios *and *susto *frequently had a personalistic dimension, in so far as emotions were frequently triggered through social relations. Notably, the majority of cases of these ailments reported among households in the complete study sample occurred in women and children, and the cause was attributed to arguments or other traumatic events. Doña Elisabeth attributed her affliction to a combination of ongoing worries about the well-being of her family, and traumatic events or conflicts emanating from social relations among her extended family, in-laws and neighbors. Over the course of the study, four of her six grandchildren experienced conditions that appeared to be life-threatening. For the two grandchildren living under her roof, Doña Elisabeth paid for a few medications and lent money for their exams during the emergency episodes when her son-in-law lacked the financial resources. She also fed her other grandchildren if they came over during lunchtime, which they frequently did since their own mothers were unable to adequately feed them. At the same time, Doña Elisabeth expressed regret that she was unable to do more for her family:

I have another daughter who lives elsewhere, she has developed a bad cough, but I cannot even visit her. I am sick and so is everyone else here. We only take a pill every now and then to maintain our illnesses. There is no money, there is no work. The family is poorly nourished. There is not enough money for all, if shoes are bought for one person, there is not money left for the rest. And it is not just here in this household...wherever you look in this community, you will find illness.

### Susana and Mariluz: The resourceful daughter versus the "neglectful" daughter-in-law

Susana, Doña Elisabeth's oldest daughter, was 22 and lived with her husband in a rustic wood dwelling at the opposite end of her parents' land. Susana cared for her three young children for up to two weeks at a time on her own while her husband drove a fruit truck for a merchant. Susana's baby daughter was chronically "*débil*" (weak), frequently experiencing respiratory ailments. After being sent away empty-handed from the government health center when the child was in respiratory distress, Susana sought care from a private physician, who diagnosed the condition as a grave case of pneumonia. Since Susana's husband was away, the physician agreed to provide the treatment on credit. Her husband paid the physician upon his return, and did so again the second time their daughter was diagnosed with the same condition. The third time it occurred, however, he became very upset.

To my field assistant and me, Susana simply reported that her husband was unhappy about the treatment cost, but that she had reproached him, saying, "Only he who does not have children does not suffer." She quickly added that her husband was a very devoted father who supported her decisions to seek private medical care for their children. "He told me, 'I will not leave my daughter to die.'" According to Doña Elisabeth, however, the young couple's argument had been intense, provoking an attack of *nervios *for Susana. Moreover, the medical care came at the sacrifice of the household's food supply:

Susana did not say anything to me, out of embarrassment, but she and the kids stopped eating because after her husband returned and paid the doctor, there was no money to buy food. His *patrones *(bosses) have not yet paid him for this month, they said they do not have the money yet. I only found out because the two older children came over here one day and told me their mother had not been feeding them. I fed them right away, and have tried to do what I can to help, but we do not have any money either. Susana does not to like to talk about her problems and will not ask for help. I do what I can, but we are suffering here too.

Notably, the sympathies and protection Doña Elisabeth expressed for her daughter did not extend to her daughter-in-law. Mariluz, age 18, lived with her two children, ages one and three, in a tiny plastic tarp house located between Doña Elisabeth and Susana. In late 2002, Mariluz's 20-year-old *compañero *(domestic partner) abruptly departed for Costa Rica, with the intention of saving money to build his family a proper home, but returned a few months later after the success he anticipated proved elusive. He was now trying to support the household by buying fruit from local farmers to resell in Managua.

Mariluz's baby son was malnourished, had a rash covering most of his body for several months, and was vulnerable to respiratory illnesses. During his last two appointments at the government health center, the medications prescribed had been ineffective. In the larger study sample, health care seekers who had resorted to private medical services reported paying up to half or more of a household's monthly income. To pay off their debt to the private physicians, households often had to take drastic measures, including neglecting the health needs of other household members, and reducing their food intake. Already purchasing food on credit, Mariluz and her *compañero *were reluctant to go into further debt for health care. When the child developed pneumonia, however, Susana and Doña Elisabeth, believing the child to be "at the point of death," persuaded Mariluz to take him to the private clinic that Susana attended, obtaining services on credit. A month later, again at her in-laws' urging, she and her *compañero *took their son to a government hospital in Managua, where he was diagnosed with a blood infection. The hospital exams were free, but the couple had to obtain the medications at a private pharmacy, and could not afford to purchase all.

The child developed another respiratory infection the following month, and Mariluz went back to the private clinic, but did not return for the recommended follow up visit because she suspected the physician of overcharging for her services. Two months later, the child developed yet another severe respiratory infection. When my field assistant and I arrived for our visit one afternoon, he was lying pale and listless in a hammock after having exhausted himself with coughing fits. Mariluz told us that her mother had warned her not to let the child die, but her *compañero's *fruit sales were doing poorly, and they had not yet paid off their debt to the private physician from the previous illness episode. A neighbor whose job provided him with medical coverage had initially offered to claim the child was his in order to get the treatment covered, but when Mariluz asked him to make the arrangements, he failed to follow through: "*El odioso *(the hateful one), he never got back to me." Unable to secure a loan from relatives or neighbors, Mariluz tried to borrow medications instead. She requested an antibiotic cough syrup from Susana, who reluctantly obliged but did not want her own children's medication to be fully consumed, nor be held liable should the boy experience a reaction to a medication that had not been prescribed for him. Mariluz then asked her brother-in-law at Doña Elisabeth's house for a couple of his anti-asthma pills to aid the child's breathing. Since the pills were inexpensive, she began to buy these for her son, explaining that while they would not cure him, she was unable to afford to buy him anything else.

Doña Elisabeth and Susana began to accuse Mariluz of "abandoning" her children, explaining that even when she did seek care, it was only because they had begged her to do so. They expressed further criticism when Mariluz decided to seek work as a live-in-housekeeper in Managua, leaving her children with her mother. Like Mariluz, many women across social classes in San Rafael worked out of economic necessity, but had to contend with competing ideologies about gender roles. Not all men approved of women working, nor did many women's female kin and in-laws; hence a woman who sought employment which required others to care for her children was vulnerable to criticism. Mariluz and other women who worked as domestic servants in the nation's capital usually received just one or two days off a month, and had to leave their children behind. Most frequently, as was the case for Mariluz, the women's own mothers took on the caregiving responsibilities during these periods. Mariluz felt that she had no alternative: "We're not surviving on my *compañero's *earnings, I have to make the sacrifice of working." She also argued that her mother actually provided better care than she could, but Doña Elisabeth disagreed: "No one can replace the warmth of a child's own mother."

Although Susana was struggling as well, she maintained her mother's stance and refused Mariluz's offer to help her obtain housekeeping work in Managua: "I told her no, because I do not want to leave my children while they are young." Relations across the households continued to weaken, with tensions at times escalating into quarrels between Mariluz and Susana. Eventually, Doña Elisabeth reported that she was no longer able to share food with Mariluz and her children since she did not even have enough to feed her own household. Mariluz, in turn, increasingly divided her time between Managua and her mother's home, minimizing her contact with her in-laws.

Although both Susana and Mariluz were very poor, and their children frequently ill, Doña Elisabeth distinguished between the caregiving efforts of her daughter versus daughter-in-law, suggesting that the latter was neglectful:

The problem is that Mariluz has abandoned this child. I do not want to speak badly about other people, but she does nothing for this boy. In the health center they do nothing if you take a child there...but even if she and her husband do not have money, they need to do what they can to treat their children. She could at least try *remedios caseros *(homemade remedies).

Notably, Doña Elisabeth also implicated both the government's health services and her own son in her criticism, yet she ultimately allocated the lion's share of responsibility for the children to their mother. Shifting to her daughter, Doña Elisabeth noted that Susana's children were also frequently ill, but unlike Mariluz, "She cures them. She is often in debt because of health treatment costs, but at least she maintains her family because she does seek treatment."

### Structural violence to the social relations of everyday life

As the daughter-in-law, Mariluz was especially vulnerable to accusations about her caregiving. With two other grandchildren already living in her home, and Susana's children coming over for food as well, perhaps, it could be argued, the strained relations between daughter-in-law and mother-in-law provided a necessary pretext for Doña Elisabeth to limit her obligations to provide for all of her grandchildren. Inability to share with others made it difficult to maintain relationships, especially when it was not always clear whether refusals to share were truly based on inability versus unwillingness. To this end, Doña Elisabeth experienced strained relations with multiple extended family members. Both her in-laws and parents became gravely ill during the study period. She had sought forgiveness from her mother for being unable to provide financial or caregiving support, but was angry with her brothers, whom she perceived as being better off, for failing to offer assistance. At the same time, she had taken to avoiding contact with her in-laws because she and her husband were unable to help them, and was no longer on speaking terms with her brother's siblings who were demanding that these obligations be shared equally.

Even as people criticized others, they frequently seemed to be aware of the ways that they themselves might be vulnerable to criticism, and Doña Elisabeth was no exception. This became especially clear during a visit shortly after Easter, when she shifted from her discussion of her family's health to tell my field assistant and me of another pain she suffered, stemming from her inability to fulfill traditional norms of reciprocity that extended beyond her household, threatening her social status as a generous relative and neighbor. Notably, even her own children had noticed this shift in her behavior, and asked her to justify her actions. As she explained:

My children asked me why I no longer give nearly as much to others as I used to, and why I did not prepare the traditional *almivar *(a dessert) for our family and neighbors during Easter last week. I told them, 'My heart has not changed, I do not need to prove my heart through my actions.' Of course, it is important to be generous, the more you give the more you will get in return. And I share our mangoes with everyone, unlike my in-laws where there is always a fight over the fruit trees. I ask God for patience, to help our neighbors and us with our economic problems. God will explain everything to us some day. We should not ask why misfortunes befall us.

Doña Elisabeth's inclusion of both her family's health and her inability to contribute to an annual Easter tradition in her interview illustrates the combined toll of economic strife and the loss of the government safety net for health care on her physical, social and psychological well-being. To whom can one turn in a time of need if everyone is experiencing distress and cannot be counted on to help? When the resources to treat illnesses within the household are not available, other social obligations can no longer be met, and one's social identity, as well as the physical body, suffers. Unlike policymakers, Doña Elisabeth did not discretely distinguish between domains such as health, welfare and social relations in her discussion of suffering; in everyday life, suffering is not "splintered into measurable attributes" [43]. For residents of San Rafael, ongoing experiences with struggles to maintain not only their basic subsistence needs, but a myriad of social obligations that symbolically affirm their ties with others, have been taxing physically, emotionally and socially.

The inability to maintain social reciprocity has placed local safety nets in jeopardy, causing the social body to suffer as well. Notably, even as household members in San Rafael recognized the constraints, they still experienced disappointment and resentment when others failed to provide the support they "should," as well as distress each time they themselves had to decline someone's request for assistance. Thus the poor experienced additional anguish through their guilt over not living up to cultural ideals, and through their frustration with others who did not do so either.

In her summaries of household members' health statuses, Doña Elisabeth alluded to multiple levels of responsibility for health care. To this end, she qualified nearly every treatment (or lack thereof) she herself administered to her kin and self with a description of the household's limited economic resources, and the lapsed responsibility of the state and "developed" countries to provide health care services, as well as her faith in God to help her family and others in her community. As she stated during one interview:

Little Kevin and Aldo both have kidney infections according to the health center exams. The children were supposed to get a blood exam as well but the health center lab did not have the materials needed to draw blood from children, nor were there any medications in stock. I do not know what is going on in this health center. Nearly all of the children suffer from this illness but there are no medications. And the children are the most delicate, they are the most ill and they cannot endure the way adults do. They are left with only prescriptions because there is no money to buy anything. I paid for their injections because my son-in-law had no money. And he is sick too, but he has no money to cure himself. Ay, we are praying to God... If only the more developed nations would at least offer discounted medications for Nicaragua..."

Dona Elisabeth's perception of multiple levels of responsibility for health care was widely shared by others in this study. While residents of San Rafael believed that their government had an obligation to provide affordable, accessible health care services, they did not absolve themselves or others of individual responsibility for health outcomes, even in cases of extreme poverty. In the social relations of everyday life, individuals were often the most visible, and hence, the most vulnerable to being held liable, both in the eyes of themselves and others. Limited capacity to live up to the expectations they and others had of themselves, and for others to do likewise, resulted in personal guilt and social conflict.

## Discussion

For households at the margins of poverty, illnesses constitute a time when the fault lines of society become transparent, revealing the conflicts between social norms and material contingencies. Of particular relevance to this case study, political economic forces, including structural adjustment policies, may render certain patterns of resort less available due to exacerbations in poverty conditions [29]. In San Rafael, the need to pay for services previously provided at no or minimal cost strained most of the study sample's household budgets to the point where all resources went to meeting basic subsistence needs, and many could not fully cover these expenses. As a result, one change widely discussed among study participants was the decreased ability of relatives to help one another in times of need, especially to provide loans or other forms of financial assistance. Relatives and neighbors did help one another with caregiving and shared medications, but tensions arose when people felt they were being asked to give more time or material resources than they could afford.

Anthropologists conducting research in Nicaragua during the 1980s - mid-1990s observed that owing to a confluence of global and local political economic factors, women's responsibility as economic providers for their children and themselves increased, as did the importance of female-centered social networks. At the same time, tensions emerged from competing gender ideologies, political strife and material constraints that impeded reciprocal exchange relations [10,13,31]. Mariluz's struggle to fulfill her obligations to be the primary caregiver for her children in the absence of sufficient economic resources resulted in a conflict that similarly affected many women her local community and nation. Following the Sandinista revolution, women were encouraged to participate in the military, and eventually acquired nearly a third of the elected positions in the Sandinista party [13]. Women became especially prominent in the workforce, replacing men who were serving in the military, and thus played a vital role in the economic sector at the national level. The absence of men also increased women's responsibility for their families, while involvement in the workforce enabled women to increase their autonomy and pressure the government to enact legislation to address the subordination and abuse women experienced in their everyday lives [31].

In many respects, however, women's status and rights changed little. While the Sandinista regime enacted some women's rights legislation, "it stopped short of transforming gender relations in the family and society" [13]. The National Opposition Union coalition that followed in the 1990s reinvoked a conservative gender ideology that emphasized traditional gender roles in the domestic and public spheres; however, the policies implemented by the regime made the fulfillment of these roles impossible for most households to achieve. In addition to further reducing the state's workforce, the new regime sought to lower the state budget by "eliminating government subsidies of food, public utilities and transportation, increasing the sales tax...devaluating the currency...reducing access to credit" and other measures that undermined households' economic security [13]. As a result, women have had to increase their labor in both the domestic and public spheres, and even then, household economies for most families have not stabilized. While the UNO government and USAID-funded school textbooks promoted "traditional family values", SAPs have done little to bolster the populace's ability to maintain family formations. Instead, the destruction of the family unit and the rise in female-headed households has been one of the damaging effects of structural violence [13].

This study illustrates, through the example of health care resources, that social relations and cultural values do not simply exist *a priori*, but arise from and change in response to particular kinds of social organization. As the national safety net for health care and other social services has been eroded, the viability of local level social formations and their ability to respond to struggles collectively have diminished as well. In San Rafael, longstanding ideals of reciprocal exchange persisted, but in conditions of poverty, expectations were often unfulfilled.

In this case study, I have examined how the social relations of health care seeking were negotiated within the broader context of intra- and inter-household interactions. As a potential safety net to offset the diminishing services and support provided by the state and paid employment, social networks could constitute a significant source of support, but could also become an additional form of stress. In their discourses, people identified multiple levels of responsibility for health care, ranging from individuals to the state, foreign nations, and God. In the social relations of everyday life, however, individuals were often the most visible, and hence, the most vulnerable to being held liable, both in the eyes of themselves and others. Limited capacity to live up to the expectations they and others had of themselves, and for others to do likewise, resulted in personal guilt and social conflict. The anguish expressed by Doña Elisabeth and her daughter-in-law Mariluz on their inability to comply with social norms, or to rely on others to meet their obligations, indexed an additional form of violence and indignity that they suffered as the state's safety net diminished. The suffering generated by people holding themselves and one another responsible for circumstances beyond their control has exposed the tension between the need for social support, versus the increasingly oppositional positioning of social network members as sources of competition for limited resources.

In sum, the social relations of San Rafael have been threatened because people can no longer rely on their social capital. The political violence experienced during the dictatorship and the war, the prolonged economic crisis, and the ongoing erosion of vital services formerly provided by the public sector have generated considerable frustration and tension among households, networks of extended kin, and neighbors. Conflicts have emerged as cultural ideals pertaining to reciprocal exchange relations and other obligations could not be met. Structural violence has, in essence, weakened the very social fabric of society, undermining the economic security, social relations and structural integrity of families and neighbors.

The study findings underscore the need for bi-lateral and multilateral agencies to take into account local needs and demands, and implement policies in a manner that respects national laws, while protecting both the physical and social well-being of individuals. In the case of Nicaragua, whose constitution still mandates that the government provide health services for the population, one of the most critical needs is to appropriately allocate resources to prioritize services for the 90% of the population who lack health insurance and for whom private sector services is prohibitively expensive, rather than overly-investing in developing the private sector [28]. As illustrated by the households focused on in this case study, a component of such prioritization would be to ensure adequate inventories of essential medications and medical supplies in public sector health centers and hospitals. In addition, policies intended to stabilize the national economy should be balanced with the needs of the populace. As noted by Muhr, for example, after Nicaragua qualified for debt relief under the Heavily Indebted Poor Countries Initiative, the $400 million released between 2004-2006 was redirected to debt repayments rather than measures to alleviate domestic poverty [18].

## Conclusions

In compliance with neoliberal structural adjustment policies mandated by multilateral and bilateral agencies, government-provided health care services have been severely restricted in Nicaragua. As the state's role in the provision of health care and other vital social services has decreased, local level social formations have been under pressure to try to bridge the gap. Yet, paradoxically, in the past people were able to help each other more precisely because a stronger safety net existed, via the state. For the households examined in this case study, longstanding ideals of reciprocal exchange persisted, but in conditions of poverty, expectations were often unfulfilled and frequently led to conflict. The resource scarcity plaguing the state has permeated civil society as well, working against cooperative efforts. Restricted access to health care, in essence, has threatened not only the physical well-being of individuals, but their social support structures as well. Bi-lateral and multi-lateral agencies need to carefully assess which services might be best provided through the public sector and strengthen them, while supporting national and local efforts to maintain both the material and social well-being of the populace.

## Competing interests

The author declares that they have no competing interests.

## Authors' contributions

LT designed the qualitative study, undertook the data collection and data analysis, and drafted the manuscript. LT is the sole author.

## Pre-publication history

The pre-publication history for this paper can be accessed here:

http://www.biomedcentral.com/1472-698X/10/9/prepub
